# Impact of elective frozen vs. fresh embryo transfer strategies on cumulative live birth: Do deleterious effects still exist in normal & hyper responders?

**DOI:** 10.1371/journal.pone.0234481

**Published:** 2020-06-26

**Authors:** Fazilet Kubra Boynukalin, Niyazi Emre Turgut, Meral Gultomruk, Selen Ecemis, Zalihe Yarkiner, Necati Findikli, Mustafa Bahceci

**Affiliations:** 1 Department of Reproductive Endocrinology and IVF Center, Bahceci Health Group, Istanbul, Turkey; 2 Department of Embryology and R&D Center, Bahceci Health Group, Istanbul, Turkey; 3 Cyprus Science University, Kyrenia, Cyprus; 4 Department of Biomedical Engineering, Beykent University, Istanbul, Turkey; IRCCS San Raffaele Scientific Institute, ITALY

## Abstract

**Background:**

Is freeze-all strategy effective in terms of cumulative live birth rates (CLBRs) in all patients?

**Methods:**

This retrospective single-center study analyzed the CLBRs of 2523 patients undergoing fresh or electively frozen blastocyst transfer cycles. In 1047, cycles, the fresh embryo transfer (ET) strategy was applied for the 1^st^ ET, whereas electively frozen ET (e-FET) was performed in 1476 cycles. Female age ≤ 37 and blastocysts frozen via vitrification were included. The patients in each arm were further stratified into four subgroups according to the number of oocytes retrieved as follows: Group A: 1–5, group B: 6–10, group C: 11–15 and group D: 16–25 oocytes retrieved. The primary endpoint was the CLBR. The secondary endpoints were the ovarian hyperstimulation syndrome (OHSS) rate and the live birth rates (LBRs) following fresh ETs and e-FETs for the first transfers.

**Result(s):**

The CLBR was similar between the fresh ET and e-FET arms in group A (35/76 (46.1%) vs 29/67 (43.3%), p = 0.74) and group B (165/275 (60%) vs 216/324 (66.7%), p = 0.091), whereas significantly higher rates were detected in favor of the e-FET arm within group C (328/460 (71.3%) vs 201/348 (57.8%), p<0.001) and group D (227/348 (65.2%), vs 446/625 (71.5%), p<0.001). The OHSS rate was also found to be higher in the fresh ET arm among group C (12/348 (3.4%) vs 0/460 (0%), p<0.001) and group D (38/348 (10.9%) vs 3/625 (0.5%), p<0.001) patients than e-FET arm. Perinatal and obstetrical outcomes were nonsignificantly different between fresh and e-FET arms. However, the birth weights were significantly lower for fresh ET, 3064 versus 3201 g for singletons (p<0.001)

**Conclusion:**

Compared with a fresh-transfer strategy, the e-FET strategy resulted in a higher CLBR among patients with >10 oocytes retrieved during stimulated cycles.

## Introduction

In vitro fertilization (IVF) and intracytoplasmic sperm injection (ICSI) cycles coupled with frozen embryo transfer (FET) have been increasingly performed worldwide. Major factors contributing to this trend are improvements in extended culture conditions and the implementation of vitrification techniques with excellent survival rates [1]. Ovarian stimulation with the use of a gonadotropin realizing hormone (GnRH) antagonist protocol, which includes triggering with a GnRH agonist, elective cryopreservation of all embryos and FET in a subsequent cycle, namely, the ‘freeze-all’ concept with segmentation of IVF/ICSI treatment, has therefore been increasingly implemented in recent years. This concept originally emerged to eliminate the risk of ovarian hyperstimulation syndrome (OHSS) [2]. A preliminary analysis of clinical studies demonstrated the strengths of the freeze-all concept, including increased maternal safety, improved pregnancy rates, decreased ectopic pregnancy rates and better obstetrical and perinatal outcomes [3]. However, emerging evidence supported an increased risk of hypertensive disorders of pregnancy in FET cycles compared with fresh embryo transfers [4]. Other adverse obstetrical and perinatal outcomes, including postpartum hemorrhage and macrosomia were reported to be increased in FET [5].

Segmentation can profoundly eliminate the risk of OHSS [6]. The second reason for the evolution of the freeze-all strategy is the negative impact of controlled ovarian stimulation (COS), which leads to supraphysiological estradiol (E_2_) and progesterone (P) levels, on endometrial receptivity [7,8]. Many molecular and histological studies supported the detrimental effect of COS on endometrial receptivity [9]. However, clinical studies validating the positive pregnancy, obstetric and perinatal outcomes of freeze-all cycles in IVF/ICSI treatments are limited.

First randomized control trial (RCT) on freeze-all strategy indicated positive clinical pregnancy outcome in normal and hyper responders [10,11]. However, they had several methodological insufficiencies and the number of patients included in these studies were limited. In addition, in a retrospective cohort study it was reported that women with prior implantation failure, a freeze-all cycle had statistically significantly higher live birth rates than fresh cycle [12]. More recently, four RCTs comparing fresh versus freeze-all cycles have been published [13–16]. In all these RCTs, the average number of oocytes retrieved was ≥12, and cleavage-stage embryos were selected in 3 of the 4 studies. In an RCT of women with polycystic ovarian syndrome (PCOS), Chen et al. reported that the freeze-all strategy increases live birth rates (LBRs) [13]. In an RCT of blastocyst transfer, Wei et al. also found the same result following blastocyst- rather than cleavage-stage transfers and advocated that blastocyst-stage transfer during an FET cycle mimics natural conception better than cleavage-stage transfer [16]. The other two RCTs did not demonstrate a significant difference between freeze-all and fresh-transfer cycles [14,15]. Better embryo selection by extended culture up to the blastocyst stage, the better survival rates of blastocysts after thawing compared to cleavage-stage embryos and the different effects of the vitrification process on intracellular dynamics may explain the different results of the RCTs [1,17].

The connection between oocyte yield and the freeze-all strategy was reported in two population-based retrospective analyses [18,19]. However, these studies had heterogeneous patient populations in terms of the ages of the women and the stages of the embryos selected for transfer. In addition, the above studies could not distinguish the electively frozen cycles from the cycles in which the freeze-all strategy was used due to the risk of OHSS or premature evaluation of P. The aim of this retrospective cohort study was to compare the CLBR following blastocyst transfers between the cycles destined for the ‘electively freeze-all’ strategy and those destined for the ‘fresh-transfer’ strategy preceding subsequent FET by the number of oocytes retrieved during the stimulated cycle.

## Materials and methods

### Study population and design

**Fig 1:**
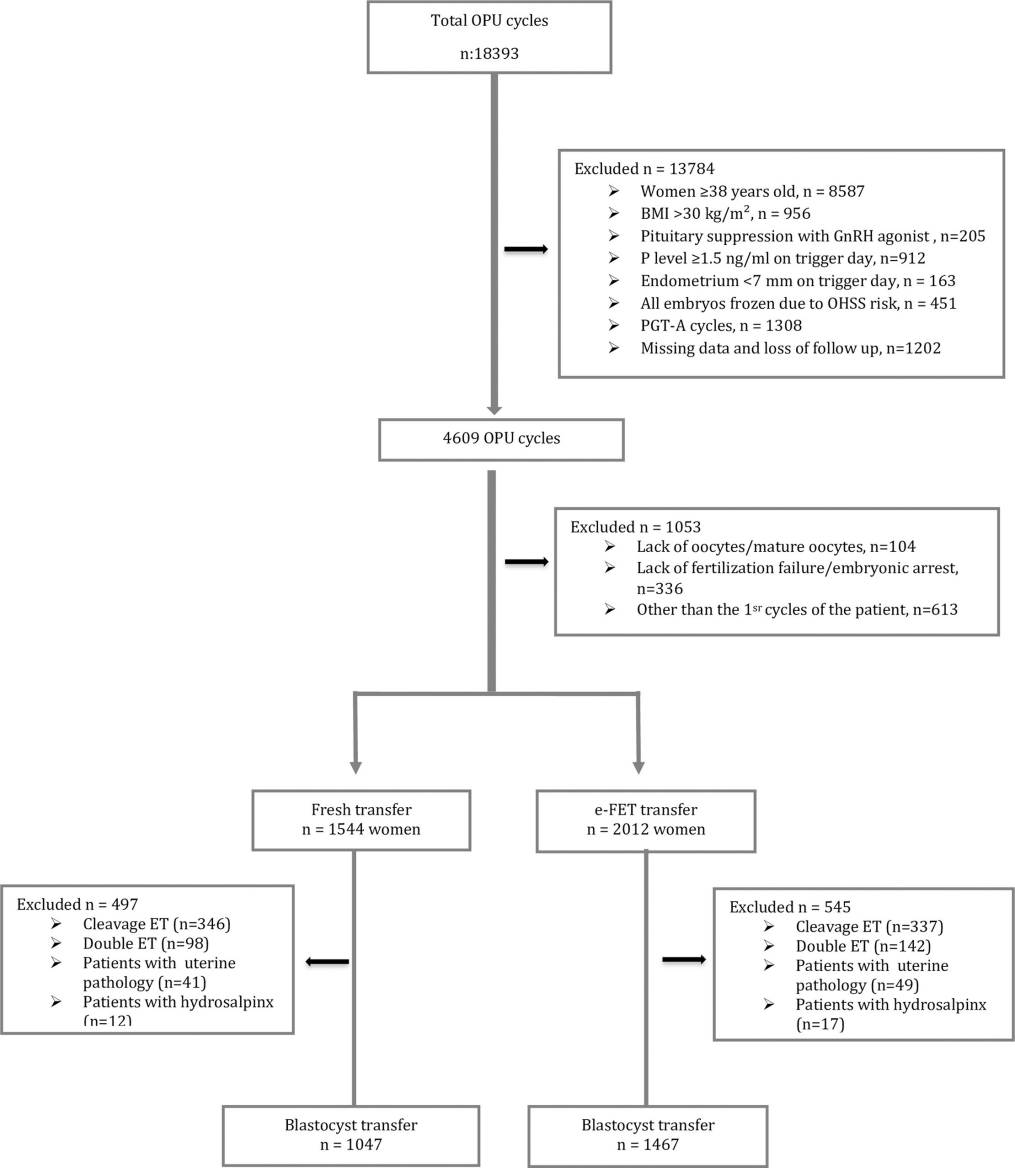
Flowchart of the study.

This was a retrospective, single-center cohort study including all women who underwent ICSI at our center between February 2012 and January 2017. Electively frozen embryo transfer (e-FET) is a common policy in our clinic and it is offered to our patients as an option (e-FET arm). The patients who don’t accept this opt undergo fresh embryo transfer (fresh ET arm). This retrospective study was approved by the Institutional Review Board of Bahceci Fulya IVF Center with a reference number of 38. The computer based data was analyzed by the permission of ethical committee. A total of 18393 oocyte pick up (OPU) cycles were analyzed, When the data set was initially filtered by the exclusion criteria 1) women aged >37 years (n = 8587) 2) body mass index (BMI) >30 kg/m^2^ (n = 956) 3) Pituitary suppression with GnRH agonist (n = 205) 4) trigger day P level ≥1.5 ng/ml (n = 912) 5) endometrial thickness < 7mm on trigger day (n = 163) 6)Freeze-all due to OHSS risk (n = 451) 7)PGT-A cycles (n = 1308) 8) missing data and loss of follow up (n = 1202), we found 4609 OPU cycles. Of these 4609 cycles, 442 cycles listed as no transferrable embryo and 613 cycles listed as the cycles other than the 1^st^ cycles of the patients. A total of 3556 OPU cycles ended with fresh and e-FET were filtered to exclude 1) cleavage ET, 2) more than 1 ET, 3) patients with uterine pathology, 4) patients with hydrosalpinx. (Fig 1). At the end, a total of 2523 patients, including 1047 patients who underwent fresh ET (41.5%) and 1476 patients who underwent e-FET (58.5%)were analyzed.

### COS, cycle monitoring and ovulation triggering

COS was initiated on day 2 or 3 of the menstrual cycle with either recombinant FSH (rFSH) (150–300 IU, Gonal-F; Meck Serono) or purified hMG (75–150 IU; Merional; IBSA). The doses of rFSH/hp-hMG were adjusted according to the patients’ antral follicle count, BMI and age. Pituitary downregulation was performed with daily administration of a GnRH antagonist starting from day 5 or 6 of stimulation. Whenever necessary, the doses of rFSH/hp-hMG were adjusted according to the ovarian response. As soon as two follicles ≥18 mm in diameter were observed during transvaginal ultrasonography (TV-USG), final oocyte maturation was triggered with 250 mcg of recombinant hCG in the fresh-transfer group and with either 250 mcg of recombinant hCG or 0.2 mg of triptorelin in the e-FET group according to the physicians’ preference. Cumulus–oocyte complexes (COCs) were collected by transvaginal aspiration 35 hours after triggering ovulation. Intramuscular (IM) P was administered daily (100 mg once a day) for luteal-phase support in all transfer cycles until the 9^th^ week of pregnancy.

### Oocyte retrieval, denudation, ICSI, embryo culture and embryo morphological assessment

In this study, in all cycles, oocyte retrieval, denudation, and ICSI procedures were performed as previously described in detail by Serdarogullari et al., (2019) [20]. After ICSI procedures, oocytes were cultured individually in a special pre-equilibrated culture dish. Single-step medium, namely, Continuous Single Culture Complete with Human Serum Albumin (Irvine Scientific, CA, USA), was used for embryo culture throughout the culture period and study. Embryo culture was performed in benchtop incubators (MIRI, ESCO Medical, Singapore). Furthermore, the developmental characteristics of each individual embryo were recorded, and blastocyst morphological evaluations were performed according to the classification of Gardner and Schoolcraft [21].

### Embryo vitrification, warming procedures and viability evaluation after warming

Vitrification and warming procedures were performed as previously described Serdarogullari et al., (2019) [20]. In this study, a commercial vitrification kit (Vit Kit ®-Freeze, 90133-SO, Irvine Scientific) for embryo vitrification and a vitrification warming kit (Vit Kit ®-Thaw, 90137-SO, Irvine Scientific) for warming procedures were used. During embryo vitrification, an open carrier device was used in all cases. Moreover, after completion of the warming procedure, the embryo was transferred to a pre-equilibrated culture dish until ET, and blastocyst grading was performed 2–3 hours after the warming procedure. Viability after warming was quantified and classified according to the percentage of surviving (100%, ≥50%, <50%, 0%) intact morphology comprising a distinguishable inner cell mass and trophoblast in a blastocyst-stage embryo and the blastocoel re-expansion ability.

### Endometrial preparation for FET

Endometrial preparation for ET involved hormone replacement therapy. Briefly, each woman was given oral estrogen (Estrofem, Novo Nordisk, Istanbul, Turkey) in a step-up regime: 4 mg/day on days 1–4, 6 mg/day on days 5–8, and 8 mg/day on days 9–12. TV-USG was performed on day 13 to measure endometrial thickness. The serum P concentration was also measured, and cycles were cancelled if the concentration was >1.5 ng/ml. Estrogen supplementation was continued at 8 mg/day, and 50-mg daily IM P (Progestan, Koçak Farma, Turkey) supplementation was started [22]. ET was performed on the 6^th^ day of P administration. Oral estrogen was continued until the 7^th^ week of pregnancy, and IM progesterone was continued until the 9^th^ week of pregnancy.

### Main outcome measures

The primary outcome of this study was the CLBR. The CLBR for the fresh-transfer strategy was defined as the number of live births deriving from fresh and frozen/warmed embryos obtained during a single ovarian stimulation cycle following fresh and frozen cycles in the same woman (the percentage of deliveries with at least one baby born from conception within 2 years after the first ET) [23]. The CLBR for the freeze-all strategy was defined as the number of live births deriving from frozen/warmed ETs obtained during a single stimulation cycle in the same women (the percentage of deliveries with at least one baby born from conception within 2 years after the first ET). All patients in the study group underwent up to 3 ETs except for 16 patients who underwent up to 4 ETs. The secondary outcomes were the LBRs (the percentage of patients with a live birth) after the first, second and third or more ETs and the OHSS rate. OHSS was classified based on the Golan criteria [24]. Only moderate and severe OHSS were recorded in the database.

Gestational age was counted from the day of embryo transfer, defined as day 18 of the menstrual cycle. Preterm delivery was defined as delivery at <37 weeks of gestation.

### Statistical analysis

The women were grouped by cycle type based on whether they had undergone the freeze-all strategy, where no embryos were transferred in the stimulated cycle and all resulting embryos were cryopreserved for transfer in subsequent cycles, or the fresh-transfer strategy, where the morphologically best embryo were transferred in the stimulated cycle and the remaining embryos were cryopreserved for future use. The patients were subdivided into 4 groups according to the number of oocytes retrieved at the end of the stimulation cycle Group A included women with 1–5 oocytes retrieved, group B included women with 6–10 oocytes retrieved, group C included women with 11–15 oocytes retrieved, and group D included women with 16–25 oocytes retrieved. These groups were selected according to number of oocytes retrieved applied by previous studies [20,25,26].

The Kolmogorov-Smirnov test was performed on continuous parameters to test whether the continuous variables followed a normal distribution, which revealed that the continuous parameters did not follow a normal distribution. Therefore, the continuous parameters are reported as the median (Quartile 1—Quartile 3) values in Table 1 and Table 2. The independent median test was used to test whether the median values of the continuous parameters were significantly different between the fresh ET and e-FET arms. The chi-squared test was used to compare patient and embryological characteristics and CLBRs between the two arms in all subgroups, and the results are reported in Table 1, Table 2 and Table 3.

**Table 1:** Patient characteristics by the type of treatment and the number of oocytes retrieved during the stimulated cycle.

	1–5 Oocytes retrieved			6–10 Oocytes retrieved			11–15 Oocytes retrieved			16–25 Oocytes retrieved		
	Fresh ET	e-FET	*p-*value	Fresh ET	e-FET	*p-*value	Fresh ET	e-FET	*p-*value	Fresh ET	e-FET	*p-*value
**No. of patients**	76	67	-----	275	324	-----	348	460	-----	348	625	-----
**Female age (years)**	32 (28–33.75)	32 (29–34)	0.515	31 (28–33)	31 (28–34)	0.394	30 (28–32.75)	30 (27–32.75)	0.953	30 (27–32)	29 (26–32)	0.145
**Male age (years)**	34 (31–37.75)	34 (31–39)	0.596	33 (30–37)	34 (30–37)	0.463	33 (30–36)	33 (30–36)	0.964	33 (30–35)	32 (30–35)	0.668
**BMI (kg/m**^**2**^**)**	23.5 (21–26)	24 (21–27)	0.539	24 (21–26)	24 (21–27)	0.967	24 (21–27)	24 (21–27)	0.413	24 (21–27)	24 (21–27)	0.590
**Type of infertility**												
**Primary**	70/76 (92.1)	55/67 (82.1)	0.072	239/275 (86.9)	264/324 (81.5)	0.071	302/348 (86.8)	414/460 (90.0)	0.154	298/348 (85.6)	553/625 (88.5)	0.199
**Secondary**	6/76 (7.9)	12/67 (17.9)		36/275 (13.1)	60/324 (18.5)		46/348 (13.2)	46/460 (10.0)		50/348 (14.4)	72/625 (11.5)	
**Reason for infertility**												
**Tubal factor**	5/76 (6.6)	6/67 (9)	0.781	23/275 (8.4)	24/324 (7.4)	0.132	25/348 (7.2)	30/460 (6.5)	0.780	15/348 (4.3)	34/625 (5.4)	0.080
**Endometriosis**	8/76 (10.5)	9/67 (13.4)		16/275 (5.8)	24/324 (7.4)		27/348 (7.8)	30/460 (6.5)		27/348 (7.8)	32/625 (5.1)	
**Uterine factor**	------	------		5/275 (1.8)	3/324 (0.9)		3/348 (0.9)	9/460 (2)		3/348 (0.9)	3/625 (0.5)	
**DOR**	45/76 (59.2)	40/67 (59.7)		16/275 (5.8)	40/324 (12.3)		-------	-------		-------	-------	
**PCOS**	-------	-------		-------	------		-------	-------		76/348 (21.8)	182/625 (29.1)	
**Male factor**	--------	-------		90/275 (32.7)	99/324 (30.6)		119/348 (34.2)	151/460 (32.8)		139/348 (39.9)	207/625 (33.1)	
**Combined male/female factors**	18/76 (23.7)	12/67 (17.9)		63/275 (22.9)	61/324 (18.8)		71/348 (20.4)	96/460 (20.9)		42/348 (12.1)	82/625 (13.1)	
**Unexplained**	-------	-------		62/275 (22.5)	73/324 (22.5)		103/348 (29.6)	144/460 (31.3)		46/348 (13.2)	85/625 (13.6)	

Values are presented as the median (quartile 1-quartile 3) or number (percentage). DOR, PCOS, and BMI denote diminished ovarian reserve, polycystic ovary syndrome and body mass index, respectively.

**Table 2:** COS and embryological characteristics of the patients by the number of oocytes retrieved.

	Oocytes 1–5			Oocytes 6–10			Oocytes 11–15			Oocytes 16–25		
	Fresh ET	e-FET	p-value	Fresh ET	e-FET	p*-*value	Fresh ET	e-FET	p-value	Fresh ET	e-FET	p-value
**No. of oocytes retrieved**	4 (3–5)	4 (3–5)	0.269	8 (7–9)	8 (7–9)	0.270	13 (12–14)	13 (12–14)	0.543	19 (17–22)	20 (17–22)	0.101
**No. of MII**	4 (3–4)	4 (3–4)	0.316	7 (6–8)	7 (6–8)	0.158	11 (9–12)	11 (10–12)	0.60	16 (13–18)	16 (14–18)	0.131
**No. of 2PN**	3 (3–4)	3 (2–4)	0.207	6 (5–7)	6 (5–7)	0.471	8 (7–10)	9 (7–10)	0.133	13 (10–15)	13 (11–15)	0.07
**Transferrable blastocyst**	1 (1–2)	2 (1–3)	0.582	3 (1–4)	3 (2–4)	0.024	3 (1–5)	4 (3–6)	0.001	4 (3–7)	6 (3–8)	0.001
**Blast quality**	**133**	**129**	**0.407**	**736**	**1019**	**0.51**	**1225**	**1954**	**0.921**	**1640**	**3676**	**0.89**
**Good**	38/133 (28.6)	38/129 (29.5)		208/736 (28.3)	286/1019 (28.1)		355/1225 (28.9)	547/1954 (28)		476/1640 (29)	1048/3676 (28.5)	
**Moderate**	88/133 (66.1)	84/129 (65.1)		487/736 (66.1)	679/1019 (66.6)		797/1225 (65)	1288/1954 (65.9)		1065/1640 (64.9)	2404/3676 (65.4)	
**Poor**	7/133 (5.3)	7/129 (5.4)		41/736 (5.6)	54/1019 (5.3)		73/1225 (5.9)	119/1954 (6.1)		99/1640 (6)	224 (6.1)	
**Endometrial thickness on trigger day**	11 (8–12.75)	11 (9–12)	0.961	10 (8–12)	10 (8–12)	0.991	10 (8–12)	11 (9–13)	0.220	10 (8–13)	10 (8–13)	0.935
**OHSS**	0/76 (0)	0/67 (0)	----	3/275 (1.1)	0/324 (0)	0.059	12/348 (3.4)	0/460 (0)	<0.001	38/348 (10.9)	3/625 (0.5)	<0.001

Values are presented as the median (quartile 1-quartile 3) or number (percentage). MII, 2PN and OHSS denote mature oocyte, 2 pro-nuclei, and ovarian hyperstimulation syndrome, respectively.

**Table 3:** CLBRs by the number of oocytes retrieved during stimulated cycles and the type of treatment.

		Fresh ET			e-FET		p-*value*
No. of oocytes retrieved	No. of women	No. of live births	CLBR (%)	No. of women	No. of live births	CLBR (%)	
**1–5**	76	35	46.1	67	29	43.3	0.740
**6–10**	275	165	60	324	216	66.6	0.091
**11–15**	348	201	57.8	460	328	71.3	<0.001
**16–25**	348	227	65.2	625	447	71.5	0.04
**Total**	1047	628	60	1476	1020	69.1	<0.001

To determine which factors affected the cumulative pregnancy outcome in all subgroups, binary logistic regression models were evaluated and are reported in Table 4. The outcome of the models was whether a patient achieved live birth. The independent factors imputed in the models at the initial step were patient age, BMI, endometrial thickness on the day of hCG administration, reason for infertility and the type of cycle (fresh ET or e-FET). However, only the statistically significant parameters were retained in the final model (Table 4).

**Table 4:** Binary logistic regression model.

No. of oocytes retrieved 11–15	B	Odds Ratio (OR)	*p-*value	95% CI for OR
Cycle type (reference: Fresh)				
e-FET	-0.597	0.550	<0.001	[0.410–0.738]
Constant	0.910	2.485	<0.001	------
**No. of oocytes retrieved 16–25**	**B**	**Odds Ratio (OR)**	***p-*value**	**95% CI for OR**
Cycle type (reference: Fresh)				
e-FET	-0.292	0.747	0.042	[0.564–0.989]
Constant	0.921	2.511	<0.001	------

Parameters: Cycle type, female age, trigger day endometrial thickness, BMI and reason for infertility.

## Results

A total of 2523 patients, including 1047 patients who underwent fresh ET (41.5%) and 1476 patients who underwent e-FET (58.5%), were analyzed (Fig 1). The patients' baseline characteristics by the number of oocytes retrieved in the fresh ET and e-FET arms are presented in Table 1. Patient age, paternal age, BMI, and reason for infertility were not significantly different between the fresh ET and e-FET arms.

Table 2 presents the COS parameters of the study population by the number of oocytes retrieved during the stimulated cycle. Comparisons between the patients in the fresh ET and e-FET arms in all subgroups (groups A, B, C and D) showed no significant differences regarding the number of oocytes retrieved, the number of mature oocytes, the number of 2 pronuclei (PN) and endometrial thickness on the trigger day. However, the number of transferrable blastocysts was significantly higher in the e-FET arm in group C (11–15 oocytes retrieved) and group D (16–25 oocytes retrieved) [4 (3–6) vs 3 (1–5), p = 0.001] and [6 (3–8) vs 4 (3–7), p = 0.001, respectively]. The embryo quality rates for fresh ET versus e-FET in all subgroups were similar.

The CLBRs by the number of oocytes retrieved during the stimulated cycle are shown in Table 3. The CLBRs were similar between the fresh-transfer and e-FET arms in group A (1–5 oocytes retrieved) [35/76 (46.1%) vs 29/67 (43.3%), p = 0.74] and group B (6–10 oocytes retrieved) [165/275 (60%) vs 216/324 (66.7%), p = 0.091]. However, in group C (11–15 oocytes retrieved) and group D (16–25 oocytes retrieved), the CLBRs were significantly higher in the e-FET arm than those in the fresh-transfer arm [328/460 (71.3%) vs 201/348 (57.8%), p<0.001] and [447/625 (71.5%) vs 227/348 (65.2%), p = 0.04 respectively]. Consistent with our hypothesis, the OHSS rate was higher in the fresh ET arm in group C [12/348 (3.4%) vs 0/460 (0%), p<0.001] and group D [38/348 (10.9%) vs 3/625 (0.5%), p<0.001] than in e-FET arm. The cycle-specific LBRs by the number of oocytes retrieved during the stimulated cycle for the first, second and third ETs are reported in S1 Table. In groups C and D, in the first ET, the LBRs were significantly higher in the e-FET arm than those in the fresh-transfer arm [group C: 45.7% (59/348) vs 58.3% (268/460), p<0.001; group D: 46.3% (161/348) vs 58.2% (364/625), p<0.001]. As expected, the LBRs for the second and third transfers were not statistically different among the groups.

To further assess potential confounding effects on the CLBRs, each group (groups A, B, C, and D) was analyzed separately in a binary logistic regression model. Maternal age, BMI, endometrial thickness on the day of trigger, reason for infertility and the type of treatment (fresh ET vs e-FET) were included in the regression model. In groups A and B, no parameters were found to be significant. However, in group C, the type of treatment, whether e-FET or fresh ET, was found to be the only significant factor favoring e-FET (OR: 0.55, 95% CI: 0.41–0.74, p<0.001) (Table 4). In addition, in group D, the type of treatment, whether e-FET or fresh ET, was found to be the only significant factor favoring e-FET (OR: 0.75 95% CI: 0.56–0.99, p<0.001).

The perinatal and obstetric outcomes are presented in Table 5. The perinatal outcomes of gestational age and preterm delivery rate were non-significantly different between fresh ET and e-FET groups. The birth weights were significantly lower for fresh ET, 3064 versus 3201 g for singletons (p<0.001). The obstetrical outcomes, were not different between the two groups.

**Table 5:** Comparison of obstetrical and perinatal outcome after the first transfers in e-FET and fresh ET groups.

	e-FET	Fresh ET	OR	RR	p-value
**Live Birth (n)**	841	487	------	------	------
**Gestational age (week ±SD)**	37.72±2.84	37.81±3.6	-------	------	0.6
**Preterm delivery (<37 weeks), n (%)**	157/841 (18.7)	87/487 (17.9)	1.055	1.035	0.715
**Term delivery (≥37 weeks), n (%)**	684/841 (81.3)	400/487 (82.1)	0.948	0.966	0.715
**Birth weight**	3201±617	3064±594	------	------	<0.0001
**Gestational hypertension, n (%)**	72/1025 (7)	42/590 (7.1)	0.986	0.991	0.943
**Gestational diabetes mellitus, n (%)**	55/1025 (5.4)	24/590 (4.1)	1.337	1.213	0.244
**Preeclampsia, n (%)**	37/1025 (3.6)	16/590 (2.7)	1.343	1.217	0.329

## Discussion

The CLBR is the preferred measure of success for IVF/ICSI procedures and allows us to better understand the real efficacy of treatment [23]. Only a few reports have compared the CLBRs of the fresh and e-FET strategies based on the number of oocytes retrieved. To the best of our knowledge, this is the first single-center cohort study evaluating the CLBRs achieved either with the e-FET strategy or the fresh ET preceding subsequent FET strategy by transferring only blastocyst stage embryos and stratified according to the number of oocytes retrieved. Our study showed that compared to the fresh-transfer strategy, the e-FET strategy resulted in a higher CLBR among patients with >10 oocytes retrieved.

It should be noted that all evaluations and comparisons in the current study was performed as “per ET”, not “per cycle” basis. In this regard, although this approach could help us to better document and compare the possible effects of COS on endometrial receptivity, the efficacy of this approach with respect to parameters at the start of the cycle could be unintentionally underestimated or ignored. Also, since COS protocols in the e-FET group could in general be executed more liberally, such approach could result an increase in the ratio of several laboratory parameters such as number of oocytes collected, fertilized, and the number of embryos developed etc. In this study, a significant difference observed on transferrable blastocysts in the e-FET group could hence be as a secondary effect of such liberal COS approaches.

Stratification based on the ovarian response may enable better prediction of IVF outcomes [27,28]. Although, patients are usually stratified according to ovarian reserve tests, stratification according to ovarian response can create more realistic predictions for IVF/ICSI outcome [29]. Furthermore, this strategy can allow definition of the discriminative parameters for choosing the fresh ET or e-FET strategy in the era of personalized medicine. Indeed, the use of a GnRH agonist trigger and a freeze-all strategy results in a drastic decrease in the OHSS risk, which is supported by our study. However, the OHSS risk is within acceptable limits with a low or intermediate oocyte yield (<15 oocytes retrieved), and overgeneralizing the results to the entire population would not be realistic.

Frozen blastocyst transfers are suggested to have some advantages, such as embryos with better implantation competency, a more receptive endometrium, and improved developmental synchrony between the embryo and the endometrium, compared to frozen cleavage-stage ETs [30]. COS, which causes supraphysiologically elevated estrogen concentrations, may alter endometrial receptivity compared to the endometrium resulting from natural cycles, and molecular, genetic and morphological studies have supported this suggestion [30–34]. Shapiro et al. was the first to report impaired endometrial receptivity in a clinical retrospective study [35]. Although alternative strategies are being proposed to improve IVF/ICSI outcomes and alleviate associated complications, the freeze-all strategy has sufficient scientific evidence supporting its improvement of the OHSS risk [6,36–38].

The first meta-analysis comparing fresh ET and e-FET was published in 2013 and included 3 RCTs analyzing normal responders and hyperresponders, and the RCTs reported higher clinical pregnancy rates in favor of e-FET [37]. In 2017, the Cochrane Library reported a meta-analysis including 1892 patients [38]. Unfortunately, this analysis did not include any trial with a poor response. The LBR after the first ET for all embryo stages (cleavage-stage embryos and blastocysts) was higher in the freeze-all group than that in the fresh ET group. However, no difference was observed between the freeze-all and fresh ET cycles regarding the CLBR per patient as the primary outcome (OR = 1.09, 95% CI: 0.91–1.31). This meta-analysis included four parallel-design RCTs. In three of these studies, cumulative results were not reported, but the authors of this study may be able to obtain these data through personal communication with the authors of the original articles. In 2018, Roque et al. reported a meta-analysis and indicated that the e-FET strategy resulted in a significantly higher LBR in hyper-responders (RR = 1.16; 95% CI: 1.05–1.28). However, the CLBR was not significantly different in the overall population (RR = 1.04; 95% CI: 0.97–1.11) [39]. Notably, the meta-analyses combined studies that randomized patients after the oocyte yield was known and excluded patients with an inadequate or excessive ovarian response. Theoretically, the freeze-all strategy is likely superior to the fresh-transfer strategy for the first ET. Subsequent transfers would not be expected to result in a higher LBR with either the e-FET or fresh-transfer strategy. Using the ‘best embryo’ in a more receptive endometrium may have an effect on the CLBR outcome. In our study, the LBR after the first single blastocyst transfer was comparable in patients from whom fewer than 11 oocytes were collected for both strategies. Conversely, the LBR after the first single blastocyst transfer showed significantly lower results in the fresh ET arm based on the increased number of oocytes retrieved (>11), which was considered to reflect the first transfer effect because in the second and third ET cycles, no statistically significant differences were found between the fresh strategy and e-FET strategy.

Although some observational studies have compared fresh ET and freeze-all strategies, no RCTs have evaluated the effects of the e-FET strategy on poor responders [39–42]. Three of these observational studies including cleavage-stage ETs did not report any benefit of using the freeze-all strategy [39–41]. Contrary to this findings, in 2017, Berkkannoglu et al. compared fresh ET and freeze-all strategies in poor responders (≤4 oocytes) with both cleavage- and blastocyst-stage embryos and reported improved pregnancy outcomes from blastocyst transfers, thus favoring the freeze-all strategy [43]. In a different point of view, Acharya et al. evaluated US national data by dividing patients into cohorts based upon the number of oocytes retrieved: high (>15), intermediate (6–14), and low responders (1–5) [18]. In low responders, the LBR was higher for the fresh transfers (25.9%) than that for FETs (11.5%) (p<0.001). Only 25% of the patients included in the study underwent blastocyst transfer, and the number of embryos transferred was not detailed in the study. The authors declared that they were unable to distinguish which embryos were electively frozen due to patient and clinical preferences and which embryos were frozen for premature luteinization, which is associated with a poor prognosis. The main strengths of our retrospective study are that e-FET was precisely defined, and patients who underwent freeze-all cycles due to an impending OHSS risk or premature P elevation (P >1.5 ng/ml) were excluded in both the fresh and e-FET arms. Although the number of patients is under power, in the poor ovarian response group (<6 oocytes retrieved) we concluded that the LBRs for the first ET and CLBRs were not significantly different for fresh ET and e-FET even after the exclusion of patients with premature P elevation. The main rationale for e-FET is the altered endometrial receptivity, which is more commonly observed in hyper-responders whose uterine environment is exposed to supraphysiological hormonal levels [31–34]. The hormonal levels after gonadotropin stimulation in poor responders are closer to physiological levels, which may explain the comparable pregnancy outcomes.

The studies evaluating the LBRs for fresh ET reported that the LBR fails to increase when more than 10–11 oocytes are retrieved [44]. Moreover, the LBR decreases when >15 oocytes are retrieved, which is an indirect indicator of impaired endometrial receptivity [44]. The available RCTs comparing fresh ET versus freeze-all policy included patients with both a good prognosis and patients with a good ovarian response (normal and high responders) [13–16]. Chen et al. included cleavage stage embryo transfers of PCOS patients in their study and reported significantly higher LBR in FET arm compared to fresh ET arm but failed to show any statistical significance with respect to CLBRs [13]. As known, PCOS patients refers to high responder group and elective freeze- all strategy is recommended worldwide to prevent OHSS, a life threatening condition, and to alleviate the harmful effects of supra-physiologic steroid hormones on the endometrium before embryo implantation. So, comparing fresh ET with e-FET must not be a conceivable approach in our current practice. Shi et al. conducted their study on normo-responder patients and compared both transfer strategies following cleavage stage embryo transfers [14]. Pregnancy outcomes after the first transfer attempts were comparable between groups. The data did not report CLBR calculation. In a similar fashion, Vuong et al. declared comparable pregnancy outcomes following cleavage stage transfers in fresh ET and e-FET groups [15]. In addition, CLBRs were also not significant at the end of the 12 months follow-up period. Most recently, in their RCTs, Wei et al. reported significantly higher LBR in FET group compared to fresh ET group following single blastocyst transfer strategy [16]. An average of 14 oocytes were collected from the participants and this was compatible to our findings in which the LBRs following fresh ET were significantly lower when the number of collected oocytes exceeds 10. Aforementioned RCTs were irrespective of different ovarian responses (the number of retrieved oocytes) in normal responders, who would have different outcomes. Similarly, a US national study evaluated the results of intermediate responders with 6–15 oocytes retrieved. However, in a retrospective analysis, Roque et al. (2017) divided normal responders into two subgroups: with 4–9 oocytes retrieved and with 10–15 oocytes retrieved [25]. They concluded that patients with 4–9 oocytes retrieved had a similar ongoing pregnancy rate in the freeze-all group. In our study, we found that the e-FET strategy is beneficial for LBRs in the first transfer as well as CLBRs in patients with >10 oocytes retrieved whereas the LBR and CLBRs in patients with 6–10 oocytes retrieved were similar in fresh transfer and e-FET group. In a parallel with the poor responders, in this group uterine environment is suggested to be less effected. As mentioned by Roque et al. (2017), our study supports dividing the normal responders into subgroups [25].

In a retrospective cohort study, the CLBR of the freeze-all strategy was similar to that of the fresh strategy when ET was carried out at the cleavage stage [45]. However, the freeze-all strategy seems to be superior if associated with cryopreservation and transfer at the blastocyst stage. The incompatible results of cleavage-stage versus blastocyst-stage transfer is unexplained. ET on day 5 may imply longer exposure of the endometrium to possible high steroid hormone levels upon implantation. In addition, the transfer of a cleavage-stage embryo into the uterus may synchronize development between the embryo and the endometrium. The effect of vitrification and warming procedure on cleavage and blastocyst stage embryos may be different. During the freezing procedure cellular metabolism slows down, active transport and ionic pumping may disrupt. Active transporting and ionic pumping systems are known to have some differences between cleavage stage and blastocyst stage embryos [46]. In addition, recovery of mitochondrial activity in the trophectodermal cells of the blastocyst is not similar to cleavage stage cells. The physiological distinctness may be taken in the consideration.

A recent survey analysis including 15937 births had reported increased rate of preeclampsia [47]. A recent meta-analysis demonstrated the same result [5]. It should be kept in mind that in these datasets the best embryos were transferred first in a fresh cycle and the second best or third best embryos transferred in FET may lead to abnormal placentation. However, the two recent RCT studies comparing fresh ET versus e-FET reported an increased risk with FET for hypertensive disorders of pregnancy [13–16]. In our study, although the preeclampsia rate is greater in e-FET than fresh transfers (3.6% vs 2.7%), there was no statistically significant difference between two groups. The number of live birth in our study is under power to analysis the preeclampsia incidence. The trend towards increasing preeclampsia should be considered. The absence of corpus luteum in endometrial preparation with hormone replacement therapy may be the reason of increased risk. Because corpus luteum does not only produce estrogen and P, it produces lots of metabolites and vasoactive products. In our study, the birth weight of the babies in e-FET group were significantly higher than fresh transfer babies. Our findings are in line with several studies and a recent meta-analysis in which a higher birth weight reported in babies born after FET compared with fresh embryo transfer [4,5].

Although this study was not prospective, we believe that inclusion of large number of patients with homogenized single center data increase its strength. There exists an ongoing debate on the obstetric and perinatal outcomes when comparing pregnancies from fresh ET and e-FET.

## Conclusion

Compared with a fresh-transfer strategy, the e-FET strategy results in a higher CLBR among patients with >10 oocytes retrieved during stimulated cycles. On the other hand, it should be noted that each center should evaluate these results according to their individualized ovarian stimulation, endometrial preparation, freezing and warming strategies.

## Supporting information

S1 Table. LBRs after the first, second and third embryo transfers (DOCX)

S1 File (PDF)

## Abbreviations

CLBR: cumulative live birth rate
ET: embryo transfer
e-FET: electively frozen embryo transfer
OHSS: ovarian hyperstimulation syndrome
LBR: live birth rate
IVF: in vitro fertilization
ICSI: intracytoplasmic sperm injection
FET: frozen embryo transfer
GnRH: gonadotropin realizing hormone
COS: controlled ovarian stimulation
E_2_: estradiol
P: progesterone
RCT: randomized control trials
PCOS: polycystic ovarian syndrome
BMI: body mass index
PGT/D: preimplantation genetic testing or diagnosis
rFSH: recombinant FSH
COC: cumulus–oocyte complexes
IM: intramuscular
PN: pronuclei
