# Supplementary material for: Impact of elective frozen vs. fresh embryo transfer strategies on cumulative live birth: Do deleterious effects still exist in normal & hyper responders?
Source: PLoS One. 2020 Jun 26;15(6):e0234481. doi: 10.1371/journal.pone.0234481 (PMC7319321; doi:10.1371/journal.pone.0234481)
Supplement: S1 Table. LBRs after the first, second and third embryo transfers — (DOCX) [file pone.0234481.s001.docx]

**Supplementary Table 1:** LBRs after the first, second and third embryo transfers.

|  | **Fresh Strategy** | **e-FET Strategy** | ***p-value*** |
| --- | --- | --- | --- |
| **Group A (1-5 oocytes)** |  |  |  |
| **1^st^ ET** |  | | |
| **No. of embryos transferred** | 1 (1-1) | 1 (1-1) | --------- |
| **LBRT** | 30/76 (39.5) | 29/67 (43.3) | 0.644 |
| **2^nd^ ET** |  | | |
| **No. of embryos transferred** | 1 (1-1) | 1 | 0.176 |
| **LBRT** | 5/14 (35.7) | 0/3 (0) | 0.218 |
| **3^rd^ ET** |  | | |
| **No. of embryos transferred** | 1 (1-1) | ------- | ------- |
| **LBRT** | 0/2 (0) | 0/0 (0) | ------ |
| **Group B (6-10 oocytes)** |  |  |  |
| **1^st^ ET** |  |  |  |
| **No. of embryos transferred** | 1 (1-1) | 1 (1-1) | ------ |
| **LBRT** | 137/275 (49.8) | 180/324 (55.6) | 0.161 |
| **2^nd^ ET** |  |  |  |
| **No. of embryos transferred** | 1 (1-1) | 1 (1-2) | 0.053 |
| **LBRT** | 23/58 (39.7) | 34/67 (50.7) | 0.214 |
| **3^rd^ ET** |  |  |  |
| **No. of embryos transferred** | 2 (1-2) | 2 (1-2) | ------- |
| **LBRT** | 5/10 (50) | 2/7 (28.6) | 0.377 |
| **Group C (11-15 oocytes)** |  |  |  |
| **1^st^ ET** |  |  |  |
| **No. of embryos transferred** | 1 (1-1) | 1 (1-1) | -------- |
| **LBRT** | 159/348 (45.7) | 268/460 (58.3) | <0.001 |
| **2^nd^ ET** |  |  |  |
| **No. of embryos transferred** | 1 (1-1) | 1 (1-1) | 0.877 |
| **LBRT** | 36/91 (39.6) | 47/90 (52.2) | 0.087 |
| **3^rd^ ET** |  |  |  |
| **No. of embryos transferred** | 2 (2-2) | 2 (1.5-2) | -------- |
| **LBRT** | 5/17 (29.4) | 9/17 (58.8) | 0.09 |
| **Group D (16-25 oocytes)** |  |  |  |
| **1^st^ ET** |  |  |  |
| **No. of embryos transferred** | 1 (1-1) | 1 (1-1) | ------- |
| **LBRT** | 161/348 (46.3) | 364/625 (58.2) | <0.001 |
| **2^nd^ ET** |  |  |  |
| **No. of embryos transferred** | 1 (1-2) | 1 (1-1) | 0.022 |
| **LBRT** | 48/109 (44.0) | 68/141 (48.2) | 0.510 |
| **3^rd^ ET** |  |  |  |
| **No. of embryos transferred** | 2 (1-2) | 2 (2-2) | -------- |
| **LBRT** | 14/26 (53.8) | 14/25 (56) | 0.877 |
