# Supplementary material for: Impact of elective frozen vs. fresh embryo transfer strategies on cumulative live birth: Do deleterious effects still exist in normal & hyper responders?
Source: PLoS One. 2020 Jun 26;15(6):e0234481. doi: 10.1371/journal.pone.0234481 (PMC7319321; doi:10.1371/journal.pone.0234481)
Supplement: S1 File — (PDF) [file pone.0234481.s002.pdf]

bahçeci
SAĞLIK GRUBU

24/01/2018

Bahçeci Fulya IVF Center

Ethics Committee

Application number: 38

Ethical approval was granted to Research and Development of Bahçeci Fulya IVF Center for the retrospective study "Comparison of cumulative live birth rates after freeze-all and fresh transfers".

Sincerely

Burçak Erzik, M.D.

Obstetrics and Gynecology

Bahçeci Fulya IVF Center

Op. Dr. Burçak Saygı Erzik
Kadın Hast. ve Doğum Uzmanı
Dip. No: 98 Ms 004

Remzi Abalı, M.D.

Obstetrics and Gynecology

Bahçeci Fulya IVF Center

Göçhan Güvenç Karlıkaya, M.D.

Obstetrics and Gynecology

Bahçeci Fulya IVF Center

Op. Dr. Göçhan Güvenç KARLIKAYA
Kadın. Hast. ve Doğum Uzmanı
58701 / 6038
